# Seronegative Myasthenia Gravis: A Rare Disease Triggered by SARS-CoV-2 or a Coincidence?

**DOI:** 10.7759/cureus.67511

**Published:** 2024-08-22

**Authors:** Beatriz Castro Silva, Miguel Saianda Duarte, Nuno Rodrigues Alves, Patricia Vicente, José Araújo

**Affiliations:** 1 Internal Medicine, Hospital Beatriz Ângelo, Loures, PRT; 2 Neurology, Hospital Beatriz Ângelo, Loures, PRT; 3 Ophthalmology, Centro Hospitalar Universitário de Lisboa Central, Lisboa, PRT; 4 Internal Medicine, Hospital CUF (Companhia União Fabril) Tejo, Lisboa, PRT

**Keywords:** post-infectious myasthenia gravis, myasthenia gravis (mg), autoimmune diseases, covid, sars-cov-2 infection

## Abstract

Myasthenia gravis (MG) results from the production of autoantibodies against the neuromuscular junction, leading to muscle weakness. Although the exact cause is not fully understood, it is known that the onset and exacerbations of MG can occur after viral infections. We present the case of a patient with no prior history of MG with new-onset proximal muscle weakness and ptosis, following SARS-CoV-2 infection, This case underscores the potential for autoimmune diseases to be triggered by SARS-CoV-2.

## Introduction

Myasthenia gravis (MG) is a neuromuscular disease characterized by the production of autoantibodies that impair neuromuscular transmission. The etiology of acute exacerbations of MG is not fully understood; however, in 30% of cases, it is triggered by infections [1]. SARS-CoV-2 virus is known to be associated with neurological manifestations and can act as a trigger for autoimmune diseases such as immune-mediated encephalitis and Guillain-Barré Syndrome [2,3].

## Case presentation

A 78-year-old independent woman with a history of Marfan syndrome, ischemic heart disease, dyslipidemia, and chronic bronchitis presented to the Emergency Department (ED) in February 2022 with a five-day history of symptoms of asthenia, bilateral ptosis more expressive on the left eye, and loss of motor strength with axial predominance, worsening by the end of the day. Her vaccinations were up to date, with no recent vaccinations.

Upon admission, neurological examination revealed marked ptosis of the left eye that worsened with fatigue but improved with the ice-pack test, mild dysphonia, and decreased proximal strength in the upper and lower limbs, with fatigability. Head CT showed no acute lesions. The patient also had a history of a mild respiratory viral infection two weeks earlier and disclosed a recent SARS-CoV-2 infection through a positive PCR test. MG was suspected, and 30mg of pyridostigmine was administered orally, resulting in an improvement in motor deficits.

**Figure 1:**
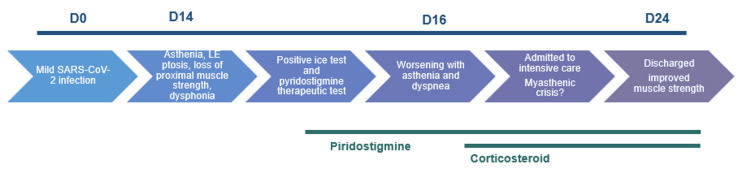
Clinical evolution D: day; LE: left eye

Three days later, the patient experienced worsening fatigue and dyspnea associated with hypoxemia, suggesting a myasthenic crisis. She was transferred to the intensive care unit (ICU) for surveillance and started on nasal oxygenation therapy (4L/min). After clinical discussion, as a trial, she was started on corticosteroids (prednisolone 40 mg/day) and pyridostigmine. The patient remained in the ICU for five days, showing favorable respiratory and neurological improvement, though ptosis in the left eye and fatigability with repetitive movements persisted. She was then transferred to the internal medicine unit and discharged after five days with pyridostigmine (Figure 1).

Electromyogram (EMG) and repetitive nerve stimulation were within normal limits, but unable to exclude neuromuscular dysfunction due to pyridostigmine administration. Anti-acetylcholinesterase receptor (AchR), Anti-muscle-specific kinase (anti-MuSK), and anti-LRP4 antibodies were negative. Neck CT showed no reliable signs of thymoma nor hyperplasic thymus. Drug etiology was excluded, as the patient was not taking any medications known to trigger MG. Decompensated seronegative MG was presumed to be due to the SARS-CoV-2 infection, with symptom improvement following therapy, leading to discharge.

## Discussion

SARS-CoV-2 infection and autoimmune activity

COVID-19 is characterized by a range of symptoms, including fever, cough, dyspnea, and myalgia. In some patients, it can lead to an inflammatory storm [4], resulting in neutrophil extracellular trap (NET) formation. NET components released into extracellular spaces can act as autoantigens, contributing to a breakdown in self-tolerance [5] and causing autoimmune activity, such as MG by a crossed reaction between anti-AchR and SARS-CoV-2.

AChR antibody-positive MG development in association with SARS-CoV-2

Initially, the association of AChR antibody-positive MG with COVID-19 was described mainly in the context of severe SARS-CoV-2 infection in ICU patients [6]. More recently, a review documented new-onset MG following SARS-CoV-2 infection [7]. There are structural similarities between the Ach receptor and the SARS-CoV-2 surface receptor, which might trigger MG. However, the occurrence of new-onset MG 5-7 days after COVID-19 positivity is unlikely; a more plausible explanation is that these patients had pre-existing subclinical MG. In contrast, the pathological mechanism related to anti-MUSK antibodies appears to be associated with a loss of immunological tolerance [6].

Seronegative MG

In this case, no antibodies were identified. The seronegativity may be due to low-affinity anti-receptor antibodies (anti-AChR, anti-MUSK, anti-LRP4) or the presence of other protein complexes not measurable in the laboratory, such as the anti-MUSK complex or anti-contractin antibodies, which mediate AChR clustering at the neuromuscular junction [8]. The underlying mechanism of seronegative MG in COVID-19 is not clearly established and requires further investigation. It might be caused by an autoinflammatory state induced by the virus, leading to an immune response mediated by cytokines, interferon-1, and NETs, which cross-react with the AchRs due to molecular resemblance, causing damage. Alternatively, it may be similar to MG anti-MUSK positive, which is related to immune exhaustion following a pro-inflammatory state [7].

There is a case in the literature describing seronegative MG post-COVID-19 infection (five months earlier) in a patient with bacterial pneumonia who was intubated and started on azithromycin and ceftriaxone [8]. To the best of our knowledge, there have been only a limited number of documented instances of new-onset MG that have been triggered by COVID-19 infection or its vaccine [9-13]. Our case reports a seronegative MG occurring two weeks after COVID-19 infection with respiratory failure, resolving after pyridostigmine and corticosteroid treatment. There are no confounding factors such as azithromycin, which is an iatrogenic cause of MG, or active infection.

Diagnosing MG was difficult due to the absence of antibodies and electromyographic changes; the diagnosis was based on the clinical exam and the improvement of symptoms after pyridostigmine. Ocular MG has some particularities, such as not being positive in repetitive nerve stimulation [14]. Another limitation in this case was the initiation of corticosteroid therapy at the onset of hypoxemia. Corticoid was started as a trial after clinical discussion due to possible worsening of the SARS-CoV-2 respiratory disease or a myasthenic crisis. Ideally, in the absence of a confounding factor, immunoglobulin should have been started in myasthenic crisis, as corticosteroids in the first two weeks can worsen symptoms [15]. At present, there have been limited instances of new cases of MG that have been initiated by COVID-19 infection and its vaccine as documented in the literature [9-13].

## Conclusions

We highlight SARS-CoV-2 infection as a potentiator of immunological activity and auto-inflammation, which could subsequently lead to the production of autoantibodies. The manifestations of autoimmune diseases can be varied and atypical, as demonstrated by this case of seronegative MG. Currently, few cases of new-onset MG triggered by both COVID-19 infection and the vaccine against this virus have been reported in the literature. To the best of our knowledge, we are the first to report a seronegative MG.
